# ANISEED 2015: a digital framework for the comparative developmental biology of ascidians

**DOI:** 10.1093/nar/gkv966

**Published:** 2015-09-29

**Authors:** Matija Brozovic, Cyril Martin, Christelle Dantec, Delphine Dauga, Mickaël Mendez, Paul Simion, Madeline Percher, Baptiste Laporte, Céline Scornavacca, Anna Di Gregorio, Shigeki Fujiwara, Mathieu Gineste, Elijah K. Lowe, Jacques Piette, Claudia Racioppi, Filomena Ristoratore, Yasunori Sasakura, Naohito Takatori, Titus C. Brown, Frédéric Delsuc, Emmanuel Douzery, Carmela Gissi, Alex McDougall, Hiroki Nishida, Hitoshi Sawada, Billie J. Swalla, Hitoyoshi Yasuo, Patrick Lemaire

**Affiliations:** 1 Centre de Recherches de Biochimie Macromoléculaire (CRBM), UMR5237, CNRS-Université de Montpellier, 1919 route de Mende, F-34090 Montpellier, France; 2 Institut de Biologie du Développement de Marseille (IBDM), UMR7288 CNRS-Aix Marseille Université, Parc Scientifique de Luminy, Case 907, F-13288 Marseille Cedex 9, France; 3 Bioself Communication, 28 rue de la Bibliothèque, F-13001 Marseille, France; 4 Institut des Sciences de l'Evolution de Montpellier (ISE-M), UMR 5554 CNRS-IRD-Université de Montpellier, F-34090 Montpellier, France; 5 Department of Basic Science and Craniofacial Biology New York University College of Dentistry, 345 E 24th Street, New York, NY 10010, USA; 6 Department of Applied Science, Kochi University, Kochi-shi, Kochi 780-8520, Japan; 7 Department of Biology and Evolution of Marine Organisms, Stazione Zoologica Anton Dohrn, Villa Comunale, I-80121 Napoli, Italy; 8 BEACON Center for the Study of Evolution in Action, Michigan State University, East Lansing, Michigan, USA; 9 Center for Developmental Genetics, Department of Biology, New York University, New York, NY 10003, USA; 10 Shimoda Marine Research Center, University of Tsukuba, Shimoda, Shizuoka 415-0025, Japan; 11 Developmental Biology Laboratory, Department of Biological Sciences, School of Science and Engineering, Tokyo Metropolitan University, 1-1 Minamioosawa, Hachiooji, Tokyo 192-0397, Japan; 12 Department of Biological Sciences, Graduate School of Science, Osaka University, 1-1 Machikaneyama-cho, Toyonaka, Osaka 560-0043, Japan; 13 Population Health and Reproduction, UC Davis, Davis, CA 95616, USA; 14 Dipartimento di Bioscienze, Università degli Studi di Milano, Via Celoria 26, Milano 20133, Italy; 15 Sorbonne Universités, Université Pierre et Marie Curie, CNRS, Laboratoire de Biologie du Développement de Villefranche-sur-mer, Observatoire Océanologique, F-06230 Villefranche-sur-mer, France; 16 Sugashima Marine Biological Laboratory, Graduate School of Science, Nagoya University, 429-63 Sugashima, Toba 517-0004, Japan; 17 Friday Harbor Laboratories, 620 University Road, Friday Harbor, WA 98250-9299, USA

## Abstract

Ascidians belong to the tunicates, the sister group of vertebrates and are recognized model organisms in the field of embryonic development, regeneration and stem cells. ANISEED is the main information system in the field of ascidian developmental biology. This article reports the development of the system since its initial publication in 2010. Over the past five years, we refactored the system from an initial custom schema to an extended version of the Chado schema and redesigned all user and back end interfaces. This new architecture was used to improve and enrich the description of *Ciona intestinalis* embryonic development, based on an improved genome assembly and gene model set, refined functional gene annotation, and anatomical ontologies, and a new collection of full ORF cDNAs. The genomes of nine ascidian species have been sequenced since the release of the *C. intestinalis* genome. In ANISEED 2015, all nine new ascidian species can be explored via dedicated genome browsers, and searched by Blast. In addition, ANISEED provides full functional gene annotation, anatomical ontologies and some gene expression data for the six species with highest quality genomes. ANISEED is publicly available at: http://www.aniseed.cnrs.fr.

## INTRODUCTION

Tunicates are a group of several thousand species of marine non-vertebrate chordates, which recent phylogenetic studies based on molecular data place as the Vertebrate sister group (1). Ascidians form the largest tunicate class and have been organized in three orders: the Phlebobranchia, Aplousobranchia and Stolidobranchia (2). These animals have fascinated developmental biologists since the pioneering works of Laurent Chabry (3) and Edwin G. Conklin (4), who showed, long before work in nematodes, that animal embryonic development could proceed with invariant cell lineages, a strategy coined ‘mosaic development’. Thanks to this very specific mode of development, ascidians and their close relatives, the appendicularians, are the only chordates whose entire embryonic developmental programme can be studied with a cellular level of resolution. Ascidian embryonic development produces tadpole-like larvae whose characteristics are shared with those of vertebrates (2), though very rare exceptions exist (5). Several studies suggest that, in spite of the simplicity, small cell numbers and peculiar mode of development of ascidian embryos, some of their developmental processes and Gene Regulatory Networks (GRN) are shared with vertebrate embryos (6,7), though it currently remains uncertain whether this similarity reflects homology or convergence.

The phlebobranchian *Ciona intestinalis* is the major model for ascidian embryonic development. In this species, whose genome was published in 2002 (8), a broad palette of molecular methods and tools have been established. *Ciona* embryos can be efficiently electroporated with DNA reporter or driver constructs (9), or microinjected with oligonucleotides or mRNAs (10). Molecular tools include morpholino oligonucleotides (11), CRISPR/Cas9 guide RNAs (12,13) and TALE nucleases (14,15) to interfere with gene function, numerous tissue specific drivers (16,17) and two collections of partial (18) or full ORF (19) cDNA clones. Thanks to these powerful tools, we have gained a very good understanding of the GRNs at work in each embryonic cell during early development (20–30). Molecular perturbations, coupled to advanced live imaging, are promising to shed light on how GRNs control the cellular processes that drive morphogenesis (31). Despite a small repertoire of fewer than 200 neurones (32,33), *Ciona intestinalis* uses the same neurotransmitters as vertebrates (34) and shows a complex stereotyped larval behavior (34). *Ciona* is thus a promising model to combine imaging, molecular perturbations and optogenetics (35), to decipher the formation and functioning of a chordate larval nervous system with cellular resolution (34).

In parallel to *Ciona intestinalis*, the ascidian *Halocynthia roretzi* also attracted attention from embryologists in Japan and Korea (2). This stolidobranchian species, which diverged from Phlebobranchia several hundred million years ago, shows a remarkably conserved embryonic cell lineage with *Ciona* (36). Interestingly, while the early developmental GRNs are generally conserved between *Halocynthia* and *Ciona*, there are some noteworthy differences (37), suggesting that some shared developmental processes have come under the control of distinct regulatory programs in the course of evolution, a process known as developmental system drift (38), and which may in part explain why some human diseases may be difficult to model in mice (39). Consistent with a high prevalence of developmental system drift in ascidians, comparison of the *cis*-regulatory logics in cell lineages conserved between *Molgula*, another stolidobranchian, and *Ciona* indicates that *cis*-regulatory sequences controlling genes with conserved gene expression patterns can sufficiently diverge to become unintelligible between species (40). Ascidians may therefore constitute privileged model organisms to study developmental system drift.

The scientific interest of ascidians is not restricted to embryonic development. Several solitary ascidians, including *Ciona intestinalis*, can undergo extensive age- and stem cell-dependent regeneration of adult brain and siphons (41), a process that now starts to be studied at the molecular level (42). The colonial ascidian *Botryllus schlosseri* has long been a model for asexual reproduction, during which adults undergo massive weekly apoptosis to be replaced by young adults through a process of stem cell-mediated budding (43). *Botryllus*, whose genome has recently been sequenced (44), is also the subject of famous studies on allorecognition and the emergence of an adaptive immune system (45,46). Finally, a large fraction of disease-associated genes are conserved in ascidian genomes (19), suggesting that ascidians can also be useful human disease models (47).

ANISEED is a sophisticated information system dedicated to the biology of ascidians, and which consists of two classes of inter-linked databases. The Developmental Browser (www.aniseed.cnrs.fr) formalizes, integrates and displays an extensive set of complementary and inter-related molecular and anatomical data for each species (48), which can be explored via four main menus giving access to functional annotations of genes and *cis*-regulatory sequences, to descriptions of anatomical entities, to gene expression data and to literature articles. Dedicated Genome Browsers complement this information by providing a visualization of genetic elements in their chromosomal context. One of the originalities of the system is to go beyond the classical hierarchical textual representation of anatomical entities found in most model organism databases and to provide a description of the shapes, neighborhood and area of cell contacts up to the early gastrula stage. The system initially focused on the embryonic development of *Ciona intestinalis*. Complete manually-curated data from 175 published articles were formalized into individual article pages (e.g. http://www.aniseed.cnrs.fr/v3/view-article.php?id = 219), which presented in—a single layout—all extracted data, irrespective of the journal in which the article was initially published.

Overall, ANISEED pursues two aims. First, it provides a service to the worldwide community of developmental biologists working on ascidians, who want to plan their next bench experiments in light of existing information. Biologists from other fields may also use it to compare results obtained in their favorite model organism to the ascidian situation. Paramount to fulfil this first aim is the simplicity and ergonomy of the user interfaces and the quality of the links to other taxa, via gene orthology or tissue homology relationships. A second—much more ambitious—aim is to integrate and formalize available molecular and anatomical data, thereby contributing to making the embryo ‘computable’ (49). Here, the challenge is to use semantic web technology to structure the data set to make it machine-understandable. Of particular importance is the inference of the architecture and dynamics of the GRNs acting in each embryonic territory, of their most likely cellular effectors and of their phenotypic output. Ascidians are promising model organisms for this second aim because of the cellular simplicity of their embryos and of the stereotyped nature of their development, based on invariant cell lineages, which should allow a complete understanding of the developmental processes acting within each cell.

The diversity of the topics studied by ascidian biologists and the recent increase in the number of laboratories working on these organisms have led to an expansion of the number of ascidian species with a sequenced genome. In parallel, novel types of genome-wide transcriptomics and epigenetic data sets have recently been developed, sometimes applicable to single cells (50–53), promising a renewed quantitative view of developmental processes. To keep up with this evolution, we refactored ANISEED to facilitate its extension to additional species, non-embryonic developmental processes and data types. The main evolutions reported here include the refactoring of ANISEED to an extended Chado schema, the redesign of all user and back-end interfaces, the improvement of *Ciona intestinalis* data, and the extension of the system to nine additional ascidian species with a sequenced genome.

## RESULTS

### Improved database architecture

The ANISEED 2010 Developmental Browser used a custom database schema, which did not make full use of ontologies, and made extensions to new data types difficult. We thus refactored ANISEED, using the highly modular and ontology-based Chado relational database schema, used by most major model organism databases (54). The choice of Chado was also motivated by the extensive set of companion tools developed by the GMOD consortium, including genome browsers (55,56), genome annotation editors (57) and workflow and analysis frameworks (58). This switch to Chado made it possible to extend the use of general ontologies. For example, ANISEED 2015 now uses qualifiers from the PATO ontology to describe morphological phenotypes (http://www.aniseed.cnrs.fr/aniseed/experiment/show_morphogen?experiment_id = 31782).

**Figure 1.**
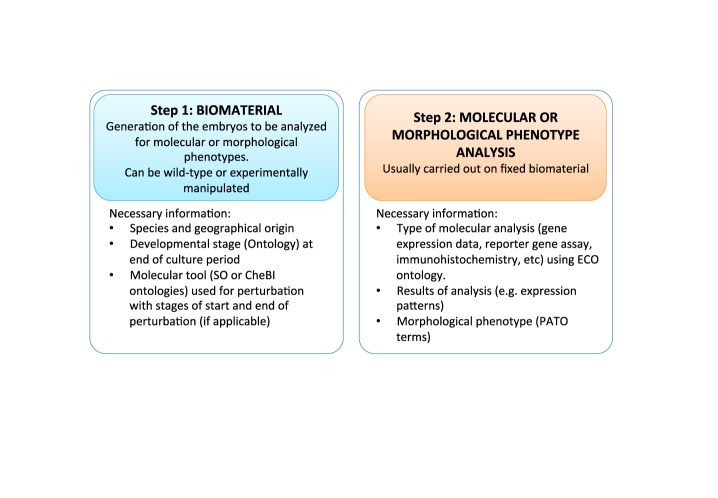
Overview of the two main structuring parts in the experiment module.

During the course of the refactoring and in order to make full use of the semantic web approach, we introduced two extensions to Chado (Martin *et al*., manuscript in preparation), which are briefly described below. First, Chado was primarily designed to represent genomic sequence-based data sets rather than the sophisticated embryological experiments performed to describe how genome information is deployed and executed in the spatio-temporal context of the embryo. We followed the Chado table philosophy to create an ‘Experiment’ module, which builds upon the existing ‘Expression’ and ‘MAGE’ modules, and links together experiments performed in parallel on control and on experimentally-treated embryos. Each experiment is described in two steps (Figure 1). The first step describes how control and experimental embryos (‘Biomaterial’, sensu MAGE module) were cultured and the type of experimental manipulation they were subjected to. Embryological perturbations are defined using a set of molecular tools generally specific for a species and are described by a sequence and a Sequence Ontology (59) term (e.g. morpholino oligonucleotides, TALE nucleases, CRISPR/Cas9 constructs). In addition, active chemical compounds (e.g. pharmacological inhibitors of signaling pathways) that cannot be described using sequence information or associated to a specific species are described using the Chemical Entities of Biological Interest (ChEBI) ontology (60). The second step of the experiment describes, using the Evidence Ontology (ECO) (61), the molecular analyses that were carried out on control or experimental embryos (e.g. *in situ* hybridization, morphological phenotype characterization). This data structure allows the extraction from the database of the specific phenotypic effect of a precisely described experimental perturbation. In the future, we will extend this strategy to associate several analyses carried out on the same biomaterial (e.g. double *in situ* hybridization; combined ISH, RNA-seq or epigenetic profiling).

The second major modification we introduced aimed at facilitating both comparative analyses between species, and the independent update of the database for each species. For this, the ANISEED Developmental Browser groups in a single database multiple copies of the same basic database schema. Each replicate schema hosts all attributes solely associated to a given species including genome, genes, transcripts, proteins, regulatory sequences, morpholinos, anatomical territory and gene expression patterns. An additional schema contains all data common to several species including articles, pharmacological reagents, and general controlled vocabulary terms such as Interpro domains, Gene Ontology, ECO, PATO, ChEBI. Each species-specific schema points to a specific Gbrowse database.

### Design of new user and biocuration graphic interfaces

**Figure 2.**
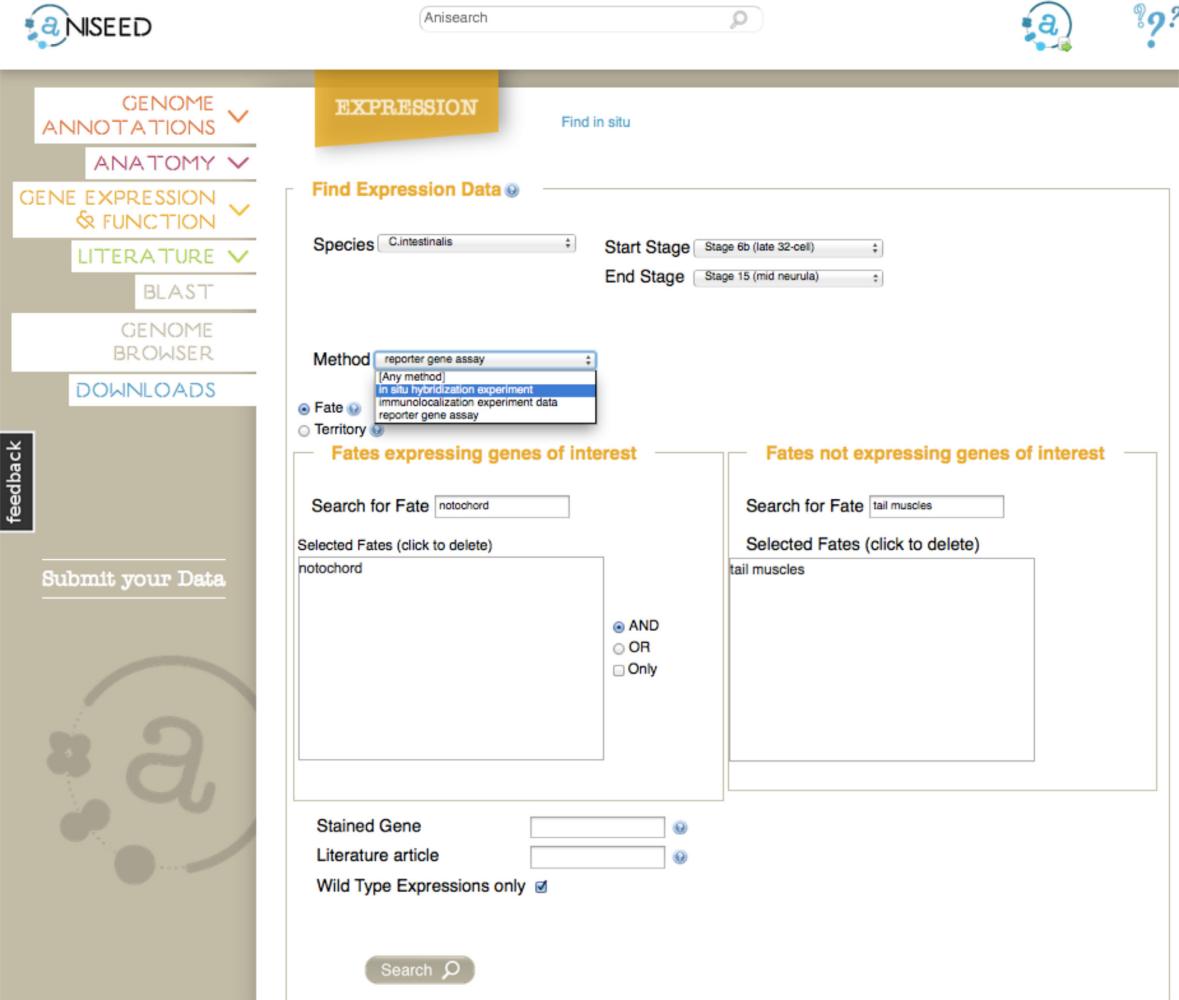
The search interface for expression patterns. Note the Anisearch field at the top of the screen, which searches the whole database for keywords, genes, anatomical entities, etc. The expression search interface permits to look for genes expressed in a set of terriories, but whose expression is excluded from another set. Searches can be restricted to a gene, or an article. Gene expression patterns in manipulated embryos can be excluded from the results.

In parallel to the above changes in the schema and database controller, the user, biocuration and back end administration interfaces were entirely redesigned with a new graphic charter, to improve their ergonomy. The landing page shows tips and news, as well as six entry tiles (“Blast”, “Genome Browser”, “Genome annotations”, “Anatomy”, “Gene expression and function" and “Literature”). Each tile gives access to a single intuitive search page (Figure 2), which centralizes all the information needed for most searches. The use of autocomplete functions in search boxes facilitates searches while ensuring the correct spelling of controlled vocabulary terms such as anatomical entities. Some of the proposed complex queries are computationally challenging, in particular searches for experiments in which a gene is expressed in a list of territories, but expression is excluded from others. Optimization of query syntax and cache usage reduced the execution time for such queries to less than 5 s.

Biocuration and user submissions are critical parameters for the success of an information system. To encourage the submission of data by users and to facilitate subsequent analysis by the biocuration team, we redesigned the biocuration graphical interfaces, again attempting to make them as intuitive as possible (Supplementary Figure 1). We also introduced a feedback button on each user interface page, through which community members may request improvements and report bugs. Finally, we created password-protected spaces to facilitate the sharing of private data sets, which has already been useful during the sequencing of *Halocynthia* and *Phallusia* genomes (see below).

### Improvement and extension of *Ciona intestinalis* data

The description of the *Ciona intestinalis* developmental programme in ANISEED 2010 was based on the original JGI assembly of the genome of a *C. intestinalis* type A individual (8) and a set of gene models built by integrating transcript models of various sources (48). ANISEED 2015 uses as reference the improved, more contiguous, KH genome assembly and a set of 15 284 manually-curated gene models (62).

**Figure 3.**
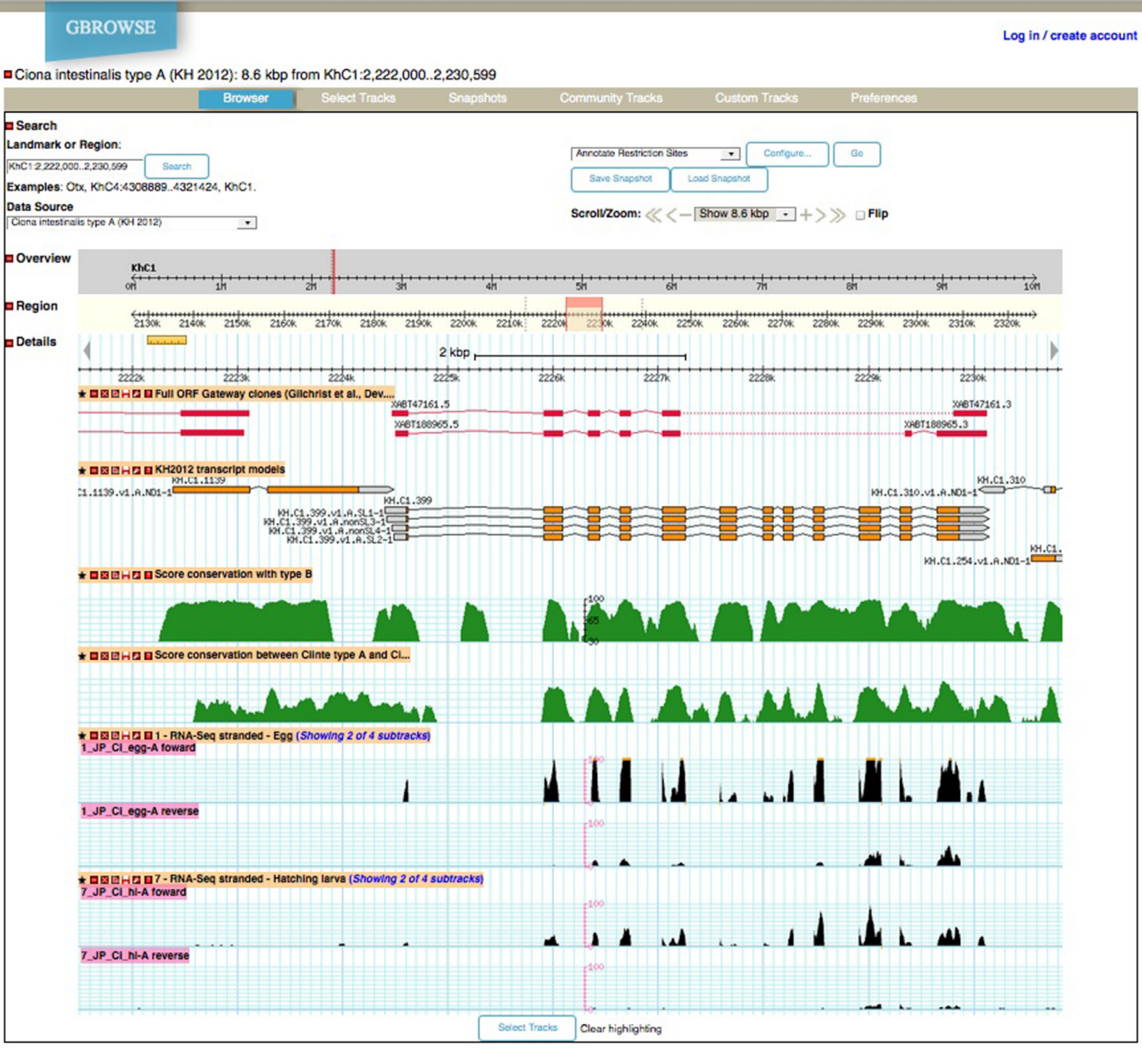
Screenshot from the ANISEED *Ciona intestinalis* type A Genome Browser.

Genetic elements can first be explored in their genomic context via the ANISEED genome browser (Figure 3). Besides gene and transcript models, this tool provides tracks for miRNAs and other non-coding RNAs, for operons (15% of *Ciona intestinalis* genes are grouped into operons) for ESTs and for RNA-seq at 6 embryonic developmental stages (stranded RNA-seq: egg, 64-cell, early gastrula, mid gastrula, mid neurula, mid tailbud and hatched larvae) and in adults (63). Additional tracks display the results of ChIP-chip data for 11 transcription factors at the early gastrula stage (20), which can be compared to the track showing the position of 875 experimentally-characterized *cis*-regulatory regions. A track also displays the local sequence conservation between *C. intestinalis* type A and *Ciona savignyi*.

Evidence has recently accumulated that *Ciona intestinalis* type B from Northern Atlantic, primarily used in Northern European and Canadian labs, and *C. intestinalis* type A from the Pacific ocean and Mediterranean sea, used by Japanese, US and Italian labs, correspond to two different species (64–67). Genomic sequences from type B individuals are sufficiently divergent from the published type A genome to impair the design of molecular tools relying on the homology of short sequences (morpholinos, TALENs, CRISPR/Cas9 guide RNAs). To overcome this difficulty, ANISEED 2015 gives access in a specific genome browser to a preliminary assembly of the *Ciona intestinalis* type B genome, which can be explored through the blast function of the system. A specific track in the ANISEED *Ciona intestinalis* type A genome browser shows local sequence conservation between the two species. Although the *C. intestinalis* type B genome assembly is too fragmented (N50 = 3.7 kb) for gene models to be built, it is sufficient to design morpholinos, TALENs or CRISPR guide RNAs for this species.

Functional *Ciona intestinalis* type A gene annotations can be explored in the Developmental Browser. Annotations are generated automatically by an updated pipeline based on the detection of conserved protein domains, on orthology relationships, and on similarity with human proteins (Table 1). The annotation process first identifies conserved domains in each protein using InterproScan 5 (68). It then computes multi-species ortholog groups between all available sequenced ascidian genomes, three vertebrates (Man, mouse and chicken) and two deeper-branching deuterostomes (the American sea urchin *S. purpuratus* and the Asian lancelet *Branchiostoma belcheri*). The multiplication of available ascidian genomes led us to use the OrthoMCL approach (69), which can simultaneously cluster orthologs from multiple species, instead of the Inparanoid-based (70) pairwise approach we previously used. Finally, the pipeline associates to each *Ciona intestinalis* gene its three most similar human proteins, identified using BlastP (e-value < 1e−15) against Swissprot. The annotation is completed by Gene Ontology terms, inherited from the domain composition of each protein using InterPro2GO (71), and from the ENSEMBL annotation of its three most similar human proteins. To avoid inheritance of irrelevant biological process GO matches (e.g. ‘limb development’, as ascidians have no limbs) to ascidian genes, these terms are filtered with a list of biological process GO terms shared between the *Drosophila*, *C. elegans* and vertebrate genomes. The functional annotation of each gene can be accessed through its ‘gene card’ page (e.g. http://www.aniseed.cnrs.fr/aniseed/gene/show_gene?feature_id=7021274). Genes annotated with specific conserved domains or Gene Ontology terms can be retrieved via the Genome annotation search page (http://www.aniseed.cnrs.fr/aniseed/gene/?choice = find_gene). Finally, to facilitate comparison with vertebrates, each *Ciona* gene can be retrieved by searching for the HUGO symbols for its best human Blast hits. Inferred gene names and symbols built according to the guidelines for the nomenclature of tunicate genetic elements (72) will be added once on-going tree-based phylogenetic work identifies accurate orthology relationships between vertebrate and ascidian genes within each OrthoMCL cluster.

**Table 1.** Some numbers about the ANISEED 2015 genome assemblies, gene annotations and gene expression patterns

Species (reference when published)	Genome (N50 scaffold size, assembly size)	Blast search	ESTs (x1000)	Embryo RNA-seq	cDNA collection	Gene models (#)	Interpro domains (mean #/gene model)	Best Blast hits to Man	# ascidian genes with human ortholog	# human genes with ascidian ortholog	GO terms (mean #/gene model)	*Cis*-reg. elements (#)	ISH patterns (#patterns; # genes)	*Cis*-reg. activity patterns
*C. intestinalis* type A (48)	5.15 Mb, 115 Mb	Yes	1205^1^	Yes	Yes (2)	15 284	2.16	Yes	7944	10 548	10.8	875	27 562; 4500	944
*C. intestinalis* type B	3.7 kb, 200 Mb	Yes	-^1^	-^1^	-^1^	-	-	-	-		-	-^1^	-^1^	-^1^
*C. savignyi* (77)	1.78 Mb, 174 Mb	Yes	84	-	-	12 165	2.05	Yes	6832	9180	10.1	-	-	-
*P. mammillata*	94.8 kb, 234 Mb	Yes	151	Yes	Yes (1)	19 508	2.01	Yes	8112	10 222	9.8	-	-	-
*P. fumigata*	11.5 kb, 249 Mb	Yes	-	-	-	-	-	-	-		-	-	-	-
*H. roretzi*	200 kb, 120 Mb	Yes	118	Yes	-	16 079	2.27	Yes	7955	10 286	10.6	-	5876; 937	-
*H. aurantium*	30.9 kb, 128 Mb	Yes	-	-	-	-	-	-	-		-	-	-	-
*M. oculata* (40)	34 kb, 160 Mb	Yes	-	-	-	15 313	2.21	Yes	9888	9638	10.8	-	-	-
*M.occulta* (40)	13 kb, 189 Mb	Yes	-	-	-	-	-	-	-		-	-	-	-
*M. occidentalis* (40)	26.3 kb, 262 Mb	Yes	-	-	-	-	-	-	-		-	-	-	-
*B. schlosseri* (44)	38 kb, 580 Mb	Yes	98	-	-	46 519^#^	0.97	Yes	9499	8269	5.7	-	-	-

^1^Some type A RNA sequences may correspond to type B individuals. #: filtered from Ref. 44 by removing monoexonic genes <1kb.

In addition to the annotation of genetic elements, ANISEED provides a detailed ontology-based description of the anatomy of developing embryos and post-metamorphic animals. In ANISEED 2010, the *Ciona intestinalis* developmental anatomy was described in 24 distinct ontologies, one for each developmental stage defined at the time. This fractionation of the anatomical description into stage-specific ontologies made it difficult to have an overview of the developmental program and to relate equivalent terms (e.g. notochord) present at successive developmental stages, and represented by different database IDs. In addition, the stages defined at the time imperfectly matched the reference *Ciona intestinalis* Hotta developmental table (73). The *Ciona intestinalis* developmental anatomy is represented in ANISEED 2015 by a single ontology, based on the Hotta developmental table, and which groups the description of developmental stages and anatomical entities. Development from the unfertilized zygote to the mature adult is temporally partitioned into 4 meta-periods (e.g. embryonic development; post-metamorphosis), 13 periods (e.g. gastrula period; juvenile) and 50 developmental stages (e.g. early gastrula). ‘Included_in’ relationships characterize the hierarchy between stages, periods and meta-periods, while ‘preceded_by’ relationships characterize their temporal sequence. This part of the ontology is conceptually similar to the Zebrafish Stage (ZFS) ontology (74). In addition 797 anatomical entities are defined and organized in a multi-dimensional tree. Each entity is characterized by: (i) its granularity (cell, cell pair or structure) via ‘is_a’ relationships; (ii) its position in the hierarchy of cells, tissues and organs via ‘part_of’ relationships, (iii) its contribution to head or tail territories via ‘belongs_to’ relationships, (iv) its temporal window of existence defined by ‘start’ and ‘end’ stages and (v) its ancestry via ‘develops_from’ relationships. Again, the structure of this part of the ontology is similar to the Zebrafish Anatomy (ZFA) Ontology (74). When possible, entity names ensure compatibility with homologous vertebrate territories (e.g. notochord, tail muscles). The larval fate of each territory is a convenient entry point in the gene expression search. To describe the larval fate of each entity, we first used ancestry relationships to associate to each territory its progeny in hatching larvae (Stage 26). To avoid defining an excessively detailed fate list, level-5 entities in the stage 26 anatomical ontology were replaced by their immediate level-4 parent, thereby defining a list of 35 larval fates. This ontology in OBO format is available in the download section of ANISEED and in the BioPortal (75) (http://bioportal.bioontology.org/projects/ASCIDIANADO). As in ANISEED 2010, the description provided by the anatomical ontology is complemented up to the early gastrula stage by a description of the spatial cell neighborhood relationships and a measure of the area of contact between neighbor cells, as these elements have been shown to be crucial predictors of early cell inductions in ascidians (76).

Using the refined genome assembly, genome models and anatomical ontology, we migrated all expression, functional perturbations and *cis*-regulatory sequence data from ANISEED 2010. Spatio temporal patterns of gene or *cis*-regulatory sequence activity are described by associating a cDNA probe or constructs to a list of anatomical entities. In addition, manual curation of 42 additional articles led to the insertion of 2,431 new gene expression patterns, 426 *cis*-regulatory sequences, 167 *cis*-regulatory sequence activity patterns and 150 molecular tools. In total, ANISEED 2015 hosts 27,707 *Ciona intestinalis* gene expression profiles by *in situ* hybridisation for around 4500 genes. These data were reported in 217 manually curated articles, include 1294 gene expression profiles in experimentally-manipulated conditions using 769 molecular tools, and 944 staining patterns describing the spatio-temporal activity of 875 *cis*-regulatory sequences in transgenic experiments. Spatio-temporal gene expression, *cis*-regulatory sequence activity and a limited number of immunohistochemistry patterns can be explored via the “Gene expression” menu of the Developmental Browser (http://www.aniseed.cnrs.fr/aniseed/experiment/find_insitu).

### Extension to additional ascidian species

ANISEED 2010 focused on the description of *Ciona intestinalis* development, with some additional data provided for the stolidobranchian *Halocynthia roretzi* based on gene models obtained by the *de novo* assembly of ESTs, as the genome of this species had not been sequenced at the time. Although the *Ciona savignyi* genome had been published (77), this species was not supported.

**Figure 4.**
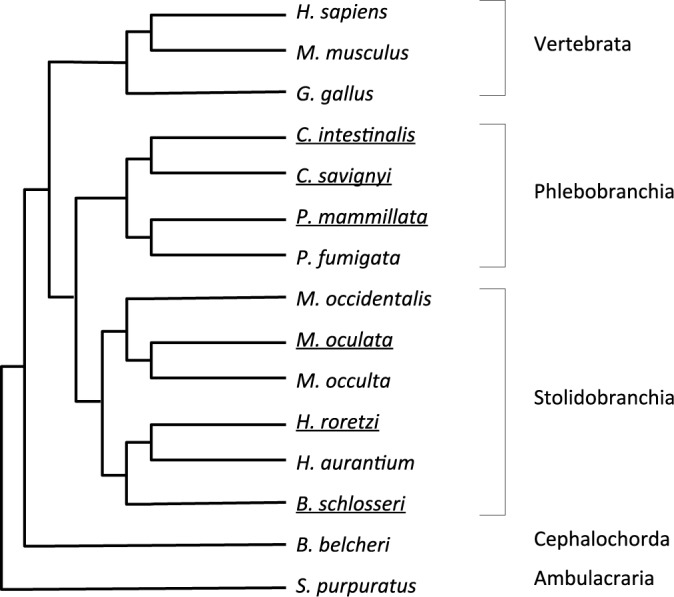
Cladogramme of the species supported with external species used for functional gene annotation purposes. The names of fully supported species with Developmental and Genome browsers are underlined.

Over the past 5 years, annotated draft genome assemblies for 7 additional solitary species have been generated (Figure 4, Table 1). The genomes of 3 species of the genus *Molgula* (Stolidobranchia) were reported (40), while those of two *Phallusia* (Phlebobranchia) and two *Halocynthia* (Stolidobranchia) species are being prepared for publication (Dantec *et al*. unpublished). In addition to these solitary species, an annotated genome draft for the stolidobranchian colonial species, *Botryllus schlosseri*, was recently published (44). ANISEED 2015 provides a Blast-searchable genome browser for each of these species, which includes for *Halocynthia roretzi* and *Phallusia mammillata* tracks for developmental RNA-seq and intra-genus patterns of sequence conservation. In addition, a Developmental Browser with detailed functional genome annotation and anatomical ontologies is provided for each of the six species with highest quality genome assemblies (*Ciona savignyi*, *Phallusia mammillata*, *Halocynthia roretzi*, *Halocynthia aurantium*, *Molgula oculata*, *Botryllus schlosseri*). Intra-ascidian orthology relationships allow the exploration of evolutionary conservation of sequences and expression patterns across ascidians. Table 1 presents for each species the genome assemblies and the genomic data and functional annotations presented by ANISEED 2015. For all new species, we followed the recently published guidelines for the nomenclature of tunicate genetic elements (72). As the new genome assemblies and gene model sets are likely to improve with time, it is important to be able to track genes across assemblies. As a first step, we created for each gene a unique gene ID, built with a uniform syntax across species (e.g. Phmamm.g00009682), and independent from both the current genome assembly and gene models. To facilitate comparisons between species, the newly supported genomes were functionally annotated using the same pipeline as *C. intestinalis*, and likewise received inferred gene names from the three most similar human genes.

In parallel to genome annotations, we created developmental anatomical ontologies for each of these new species (downloadable OBO files from ANISEED and from the BioPortal). Thanks to the exceptional conservation of embryonic cell lineages and morphogenetic processes between distantly-related solitary ascidian species (2,40), the embryonic ontologies for the different species are quasi identical, thereby facilitating comparison of their developmental programmes. Based on previous work, we considered that all solitary phlebobranchian ascidians share a common anatomical ontology. In consequence the anatomical ontologies of *Ciona intestinalis*, *Ciona savignyi* and *Phallusia mammillata* are identical. With a few documented exceptions (e.g. b8.17 cell lineage (36); delayed division of A7.6 cells in *Halocynthia* *roretzi* (48)) the stolidobranchian *Halocynthia* cell lineages are identical to the *Ciona* ones. The *Halocynthia roretzi* anatomical ontology was therefore generated from the *Ciona intestinalis* ontology, by targeted modification of the few lineages known to differ between the two species. This ontology was used to annotate 5876 wild-type expression patterns for 937 *Halocynthia roretzi* genes, using the same annotation strategy as *Ciona intestinalis*. This ontology was then considered to describe the development of all solitary stolidobranchians, including *Molgula* species. Colonial ascidians reproduce both sexually and asexually, and *Botryllus schlosseri* is currently the leading model organism for the study of asexual reproduction. ANISEED adopts the recently published BODA (*Botryllus* Ontology of Development and Anatomy) (65) to describe asexual development. In the future, it will be important to extend this ontology to the embryonic period to best compare these two programmes that give rise to essentially the same adult form. This will require substantial modification of existing solitary ascidian ontologies, as colonial ascidians produce larvae with much larger cell numbers than solitary ascidians.

### Computing ascidian development: internal reasoning engines and data accessibility

ANISEED's new architecture and data structure is a significant step toward the aim of making ascidian data computable, as the quasi-exclusive use of ontologies to describe data opens the way to semantic reasoning. To allow scientists to go beyond the current Graphical User Interface (GUI) and to reason on the data set, the download section of the system contains parsable formatted text or XML files for most data, including expression data available under the MISFISHIE format (78). To further improve data accessibility, we will provide the community with ways to dynamically extract targeted subsets of the data. We will first implement a Biomart server to retrieve complex data sets without the need to write complex SQL queries. A dedicated Application Programming Interface (API) will complement the system.

Semantic reasoning can also be used internally. For instance, ANISEED 2010 used a rules based reasoner to provide for each gene a precomputed list of upstream regulators and downstream targets in each tissue, based on the comparison of expression patterns in wild type and morphant conditions, and on *cis*-regulatory sequence analysis (48). We are currently reimplementing a similar gene regulatory network inference system, which with time will integrate additional data, including transcription factor occupancy (20), open chromatin maps (79), or phylogenetic foot-printing (80).

### NISEED, a generic system applicable to other taxa

With the advent of cheap genome sequencing, an increasing number of non-vertebrate species, in particular marine invertebrates, are accessing the status of emerging model organism in the field of developmental biology. The communities working on these species are however often small, precluding the development of specific computational infrastructures necessary to organize molecular embryological data.

ANISEED is the ascidian implementation of a generic system, NISEED, which can be easily adapted to any taxon for which a suitable anatomical ontology has been developed. The use of a software architectural pattern and cascading style sheets (CSS) structures the code thereby facilitating the customization of existing interfaces and the creation of new ones. The complete source code and installation instructions for the database, the user interfaces and extensive biocuration interfaces are available under terms of the GNU General Public License and can be obtained upon request to contact@aniseed.cnrs.fr.

### Future challenges: biocuration and integration with imaging data sets

Our main aim over the past 5 years was to refactor the system to professional standards and to increase the coverage of species to most ascidian orders. The version of ANISEED described here largely fulfils these aims and the project is now entering a new phase.

The first challenge is to extend the biocuration effort. The extension of the system to novel genomic data sets, in particular RNA-seq, ChIP-seq and associated epigenetic data sets, and *in vitro* DNA-binding specificities of transcription factors will necessitate a reflexion on how to best process and formalize these data. Biocuration will also be needed to enter into the system a large body of scientific articles, mostly describing studies carried out in *Ciona intestinalis*, *Halocynthia roretzi* and *Botryllus schlosseri*. The current biocuration pipeline consists in entering data by authors or dedicated biological annotators, followed by data verification by the system's biocurator. Entering data from the literature is much simplified when authors follow the tunicate nomenclature guidelines (72) and the Article Minimum Information Standard for tunicates (48), indicate in materials and methods the gene model IDs and the precise cDNA clones used to perform *in situ* hybridization experiments, and follow the new nomenclature for transgenic constructs (72). Priority will continue to be given to articles reporting the effect of gene loss or gain of function on the developmental programme, taking into account the willingness of authors to contribute to the insertion of their work. Several laboratories are now actively entering data from their publications, a process that will continue to be rewarded by an authorship in ANISEED update papers.

The second main challenge will be to integrate detailed molecular descriptions of the developmental programme with upcoming live imaging data sets. Some ascidians have either translucent (*Ciona*) or transparent (*Phallusia*, *Ascidiella*) embryos that are ideally suited to whole embryo imaging approaches using confocal or light-sheet microscopy. Such approaches coupled to semi-automated or automated segmentation of cell membranes have already been applied to cleavage and gastrula embryos (76,81), to tailbud embryos (82) and to individual tissues (83). ANISEED 2015 already represents cell neighborhood and areas of contact up to the early gastrula stage. The description of later stages with cellular resolution will solely require the adaptation of the current anatomical ontologies to reach cellular resolution beyond the gastrula stages. Finally, it will be important to develop powerful 4D visualization tools that will replace our previous 3DVE viewer (76).
